# Evaluation of the effectiveness of teledentistry on diagnostic accuracy and treatment planning among Jordanian dentists

**DOI:** 10.3389/fdmed.2025.1705072

**Published:** 2026-01-15

**Authors:** Sabha Mahmoud Alshatrat, Majd Alsaleh, Jumana M. Sabarini, Tamadur Mahmoud Falah, Yousef Saleh Khader, Alaa Fawwaz Dalky, Bayan Mahasneh, Abedelmalek Kalefh Tabnjh

**Affiliations:** 1 Department of Applied Dental Sciences, College of Applied Medical Sciences, Jordan University of Science and Technology, Irbid, Jordan; 2 Department of Pediatric Dentistry, University of Illinois Chicago, Chicago, IL, United States; 3 Consultant of Pediatric Dentistry, Arabella Dental Center, Irbid, Jordan; 4 Department of Public Health, Community Medicine, Jordan University of Science and Technology, Irbid, Jordan; 5 Department of Health Management and Policy, Faculty of Medicine, Jordan University of Science and Technology, Irbid, Jordan; 6 Department of Cariology, University of Gothenburg, Gothenburg, Sweden; 7 Deparment of Pathology, Saveetha Medical College and Hospital, Saveetha Institute of Medical and Technical Sciences, Chennai, India

**Keywords:** accuracy, dentist, diagnosis, Jordan, teledentistry

## Abstract

**Purpose:**

To assess the diagnostic accuracy and treatment planning agreement among Jordanian dentists when using teledentistry.

**Methods:**

Thirty children underwent dental examinations. Standardized intraoral photographs and brief case histories from pediatric patients were compiled into clinical case scenarios. Eight representative cases were selected and presented in a Google Forms survey to licensed dentists in Jordan. Participants reviewed the cases, providing clinical diagnoses and proposing treatment plans. Responses were analyzed to determine diagnostic accuracy and agreement on treatment planning.

**Results:**

Diagnostic agreement was highest for cases with distinct clinical presentations. Case #5 (early childhood caries) showed the highest agreement at 92.1%, followed by Case #3 (avulsion; 91.1%) and Case #6 (ectopic eruption; 85.1%). Treatment planning agreement followed a similar pattern. The highest concordance was reported for Case #4 (molar-incisor hypomineralization; 64.4%) and Case #7(Functional class III/anterior crossbite 59.4%).

**Conclusion:**

Teledentistry enables high diagnostic accuracy among Jordanian dentists, especially in pediatric cases with well-defined presentations. However, the observed variability in treatment planning highlights the need for standardized clinical guidelines and targeted professional development to optimize teledentistry's integration into routine dental care.

## Introduction

The rapid evolution of digital health technologies has significantly transformed healthcare delivery worldwide, with teledentistry emerging as a pivotal innovation to enhance access, efficiency, and patient outcomes in oral healthcare (1). Teledentistry, which utilizes telecommunications to deliver preventive, diagnostic, and therapeutic dental services remotely, gained considerable momentum during the COVID-19 pandemic, when minimizing physical contact became a priority (2). It facilitated consultations, triage, and treatment planning in various clinical settings and even enabled communication among dental professionals via social media platforms (15).

Globally, teledentistry has demonstrated the potential to improve access to care, especially among underserved populations, by reducing costs and expanding the reach of services (3). Systematic reviews consistently report high diagnostic accuracy and patient satisfaction with teledentistry, showing comparable performance to traditional face-to-face consultations (3, 4). These findings support its growing role in facilitating timely diagnoses and effective treatment planning, particularly in pediatric and community health contexts (4).

In Jordan, a country facing a growing population and disparities in healthcare access between urban and rural regions, teledentistry offers significant promise (5, 15). During the COVID-19 pandemic, Jordanian dentists utilized teledentistry to manage patients remotely, particularly for antimicrobial prescribing and diagnosing oral infections through professional networks (15). However, despite its adaptive use during the pandemic, teledentistry has not been widely adopted or systematically integrated into the national healthcare system (5, 6).

The widespread implementation of teledentistry in countries like Jordan faces several significant barriers. Limited technological infrastructure, particularly in rural areas, restricts access to reliable internet and telecommunication systems, which are essential for delivering remote care (7). Additionally, low levels of digital literacy among both dental professionals and patients can hinder the effective adoption of digital tools. Financial constraints, including the initial costs of establishing teledentistry platforms and uncertainty about reimbursement policies, further impede integration. Regulatory and legal challenges, such as the absence of national guidelines, concerns over patient privacy, and data security issues, also present obstacles (4). Cultural factors, including patient and provider skepticism regarding the quality and reliability of virtual care, may reduce acceptance. Finally, the inherent clinical limitations of teledentistry, which preclude hands-on procedures, restrict its use to consultations, diagnostics, and follow-ups. Addressing these barriers is critical to leveraging teledentistry's potential to improve oral healthcare access and equity in Jordan (7).This study aimed to assess the diagnostic accuracy and treatment planning agreement of Jordanian dentists using teledentistry, with a focus on pediatric cases.

## Materials and methods

This cross-sectional study was approved by the Institutional Review Board (IRB) at the Jordan University of Science and Technology (JUST) (IRB Reference: 1/161/2023) and conducted in accordance with the Declaration of Helsinki (18). Informed consent was obtained from all participants.

### Study population and recruitment

The study population consisted of licensed dentists practicing in Jordan. Participants were recruited using voluntary response sampling through professional social media platforms, including Facebook and WhatsApp, where the survey link was shared within dental practitioner groups. All actively practicing dentists were eligible to participate, regardless of specialty or years of clinical experience. Because the survey link was disseminated through social media platforms rather than a predefined sampling frame, the total number of dentists who received the invitation could not be determined; therefore, an exact response rate could not be calculated.

A formal sample size calculation was not conducted, as the study was exploratory in nature. However, recruitment efforts aimed to obtain a diverse sample with variation in clinical experience, workplace settings, and academic backgrounds to assess diagnostic consistency across different practitioner profiles.

### Survey structure

The survey was administered via Google Forms and consisted of two main sections: (1) Demographic and Professional Information. Participants reported their age, gender, qualifications, years of clinical experience, workplace setting, device used to complete the survey (e.g., laptop or phone), and average daily internet usage for professional purposes. (2) Clinical Case Evaluations Participants evaluated eight standardized pediatric dental cases. Each case included a brief patient history and 6–7 intraoral photographs. Dentists were asked to provide open-ended responses for both diagnosis and treatment planning.

### Case selection and preparation

The clinical cases were drawn from examinations of 30 pediatric patients aged 3–8 years at a pediatric dental clinic in Irbid, Jordan. Parental consent was obtained for the use of intraoral photographs. All images were anonymized and captured under standardized lighting conditions using an iPhone 11 and intraoral mirrors to ensure consistency across frontal, lateral, occlusal, and case-specific views.

**Figure 1:**
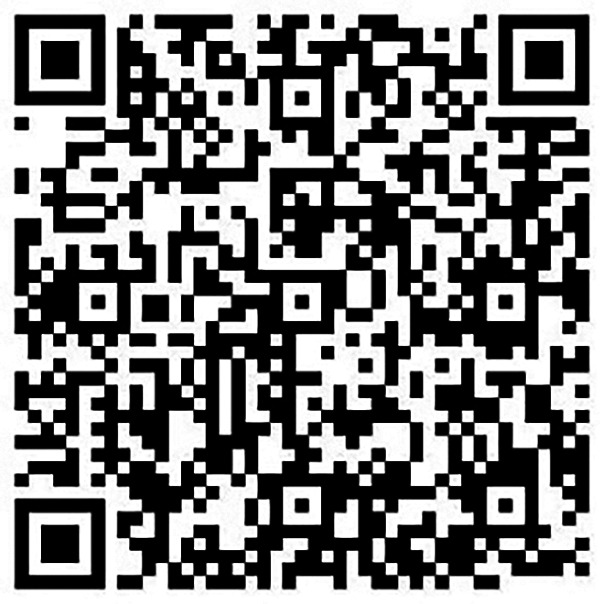
Access to full Set of clinical cases.

Two board-certified pediatric dental specialists, each with over 10 years of clinical experience, independently reviewed the 30 cases and selected eight based on the following criteria: clarity and relevance of diagnosis, high photographic quality, clinical representativeness, and absence of duplicate or ambiguous findings. Disagreements were resolved through discussion until consensus was reached. Inter-rater reliability was not calculated. In this study, the primary objective was to report the percentage of correct diagnostic and treatment decisions, which reflects the accuracy of the system within a dental context. Because the focus was on the accuracy of diagnosis and treatment outcomes—rather than agreement between two human coders—an inter-rater reliability assessment was not applicable to our research design. Our aim was to evaluate system performance, not to conduct a reliability test between human raters. Because the specialists were not blinded to the study's objectives, potential selection bias is acknowledged as a limitation. To minimize participant fatigue, only eight cases were included. These eight cases were formatted into a standardized template using Canva and embedded directly into the survey. A QR code (Figure 1) linking to all eight cases was also provided to ensure full transparency and facilitate replication and external review.

### Data coding

Open-ended responses for diagnosis and treatment planning were coded into predefined categories based on standard pediatric dental terminology and treatment protocols. This coding framework is provided in the Supplementary Documents.

### Statistical analysis

Data were analyzed using IBM SPSS Statistics version 24. Descriptive statistics (frequencies and percentages) summarized diagnostic and treatment agreement per case.Overall diagnostic and treatment accuracy scores were calculated as the percentage of correctly diagnosed and managed cases out of eight scenarios. Associations with two-category variables (gender, qualification, experience, teledentistry) were analyzed using independent-samples *t*-tests, and differences across age groups were assessed using one-way ANOVA. Statistical significance was set at *p* < 0.05.

## Results

A total of 101 dentists participated in this study, and among them, 100 dentists answered all the cases and were included in the final score. Of the respondents, 32.7% were aged 25–34 years, 29.7% were 35–44 years, and 23.8% were 45–54 years. Most participants were female (70.3%). Regarding professional qualifications, 46.5% specialized in pediatric dentistry and 32.7% were general practitioners. Smaller groups included orthodontists (5%), specialists in oral medicine/radiology/pathology (4%), and others such as public health dentists, periodontists, prosthodontists, and endodontists. Concerning clinical experience, 24.8% had been practicing for 1–5 years, 17.8% for 11–15 years, and 23.8% for more than 20 years. More than half of the participants (52.5%) had no prior experience with teledentistry, while 35.6% reported 1–4 years of experience and 12.4% had more than 4 years. Nearly half of the sample worked in the private sector (45.5%), followed by those employed in the Royal Medical Services (18.8%) (Table 1).

**Table 1:** Demographic and professional characteristics of participating dentists.

Characteristics	Frequency (*n*)	Percent (%)
Age (year)		
<25	6	5.9
25–34	33	32.7
35–44	30	29.7
45–54	24	23.8
55+	8	7.9
Gender		
Male	30	29.7
Female	71	70.3
Qualification		
Pedodontics	47	46.5
General practitioner	33	32.7
Orthodontics	5	5.0
Oral medicine/radiology + Pathology	4	4.0
Public Health, Endodontics	4	4.0
Periodontics + Oral surgery	4	4.0
Prosthodontics	4	4.0
Years of experience		
<1	6	5.9
1–5	25	24.8
6–10	17	16.8
11–15	18	17.8
16–20	11	10.9
20	24	23.8
Years of experience in teledentisry		
0–1	53	52.5
1–4	36	35.6
5–8	7	6.9
9–12	5	5.0
Participant's workplace		
Academic Institution	18	17.8
Ministry of Health	18	17.8
Private Sector	46	45.5
Royal Medical Services	19	18.8

Descriptive analysis showed that diagnostic accuracy scores ranged from 0% to 100%, with a mean of 72.8% (SD = 20.7). Treatment accuracy scores ranged from 12.5% to 87.5%, with a mean of 48.1% (SD = 17.0). Diagnostic accuracy was not significantly associated with age (*p* = 0.809), years of clinical experience (*p* = 0.477), or previous exposure to teledentistry (*p* = 0.554). Treatment accuracy similarly showed no significant association with age (*p* = 0.900), years of practice (*p* = 0.797), or teledentistry experience (*p* = 0.829). In contrast, both gender and qualification were significant predictors of performance. Female dentists demonstrated significantly higher diagnostic (76.2% vs. 64.6%, *p* = 0.010) and treatment accuracy (51.1% vs. 41.2%, *p* = 0.007) than male dentists. Pediatric dentists achieved the highest performance overall, with markedly greater diagnostic (82.7% vs. 64.1%, *p* < 0.001) and treatment accuracy (54.3% vs. 42.8%, *p* < 0.001) compared with dentists from other specialties (Table 2).

**Table 2:** Diagnostic and treatment accuracy scores by dentist characteristics.

Variable	*n* (*N* = 100)	Diagnosis Accuracy Mean (%)	SD	*p*-value	Treatment Accuracy Mean (%)	SD
Age	—	—	—	0.809	—	—
<35	38	74.2	17.6	—	47.3	15.1
35–44	30	72.9	20.8	—	49.2	15.7
>44	32	70.9	24.4	—	48.2	20.5
Gender	—	—	—	**0** **.** **010**	—	—
Male	30	64.6	18.8	—	41.2	17.7
Female	70	76.2	20.6	—	51.1	15.9
Qualification	—	—	—	**<0** **.** **001**	—	—
Pedodontics	47	82.7	14.4	—	54.3	15.9
Others	53	64.1	21.5	—	42.8	16.2
Years of Experience	—	—	—	0.477	—	—
≤10 years	47	74.3	18.2	—	48.6	15.7
>10 years	53	71.4	22.8	—	47.7	18.3
Experience in Teledentistry	—	—	—	0.554	—	—
No	53	71.6	22.1	—	47.8	18.2
Yes	47	74.1	19.1	—	49.0	15.5

*p* ≤ 0.05 was considered statistically significant.

**Figure 2:**
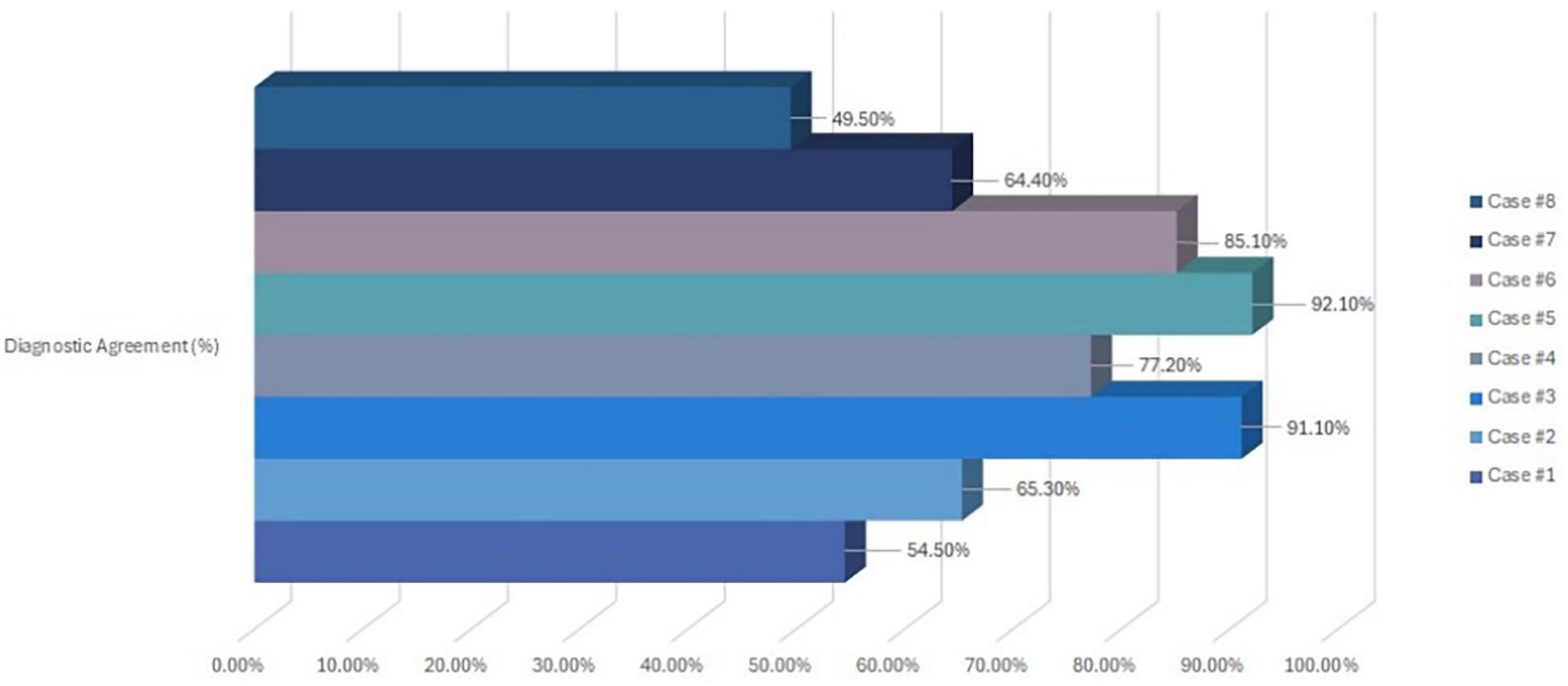
Diagnostic agreement among participating dentists for each dental case.

**Figure 3:**
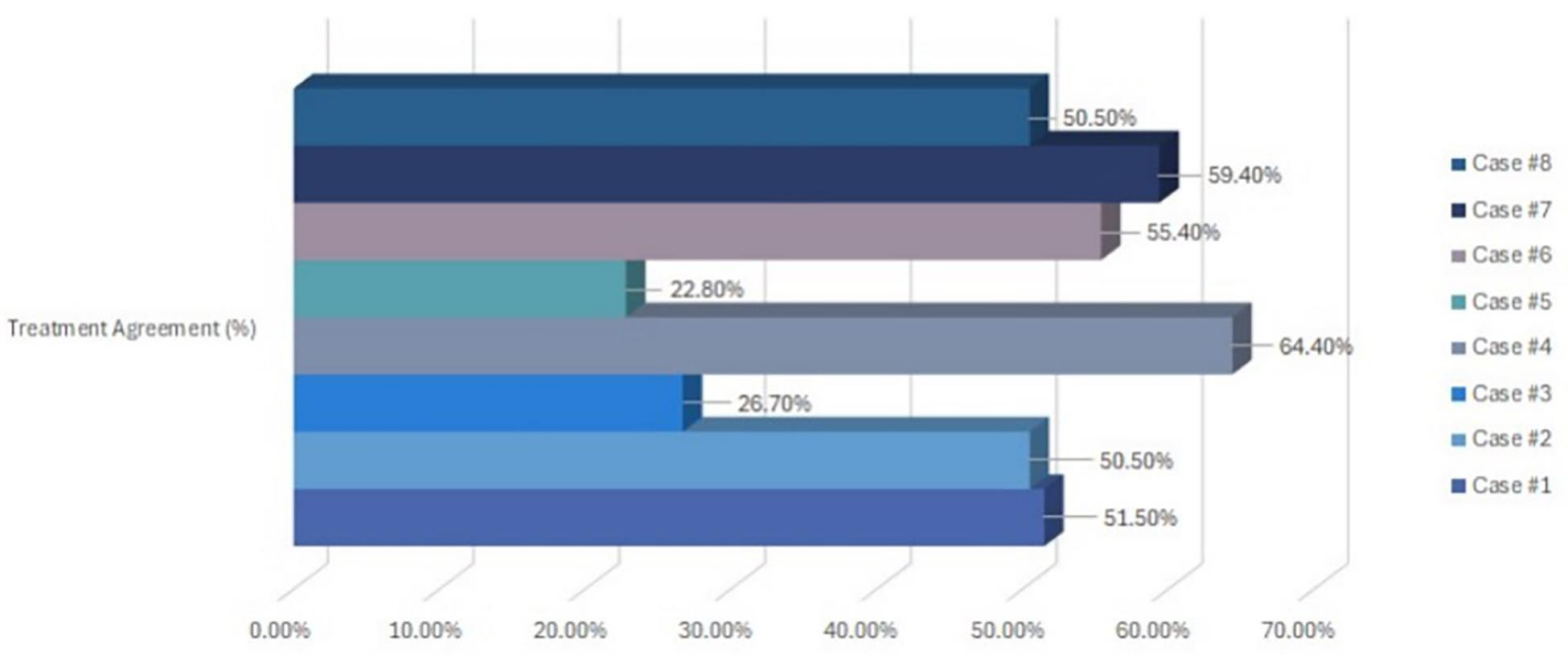
Treatment agreement among participating dentists for each dental case.

Table 3 presents the diagnostic and treatment responses across the eight pediatric dental cases. Overall diagnostic and treatment agreement levels are summarized in Table 4 and Figures 2, 3. Diagnostic agreement was strongest for cases with clear and distinctive clinical findings. Case #5 [early childhood caries (ECC)] achieved the highest diagnostic consensus (92.1%), followed by Case #3 (avulsion; 91.1%) and Case #6 (ectopic eruption; 85.1%). Moderate agreement was observed for Case #2 (abscess; 65.3%) and Case #7 (functional Class III crossbite; 64.4%). Case #8 (molar hypomineralization) showed the lowest diagnostic agreement (49.5%), suggesting greater variability in identifying subtle enamel defects (Figure 2).

**Table 3:** Cases summary: diagnosis and treatment plan as determined by a gold standard specialist and the distribution of diagnoses and treatments selected for each case by the participants.

Cases	Frequency (*n*)	Percent (%)
Diagnosis for case #1:		
Fissured tongue	17	16.8
Candida/thrush/fungal^a^	55	54.5
Geographic tongue	14	13.9
Don't know	1	1.0
Other	14	13.9
Treatment for case #1:		
Oral hygiene improve/reassurance/follow up/mouthwash/avoid certain food/palliative care/nothing/no treatment	39	38.6
Antifungal/nystatin/Miconazol/fluconazole^a^	52	51.5
Antibiotic/treat infection	3	3.0
Stop antibiotic	2	2.0
Don't know	3	3.0
Corticosteroids	2	2.0
Diagnosis for case #2		
Abscess/necrotic/infection/fistula^a^	66	65.3
Pulpitis	5	5.0
Caries/cavity/ECC	17	16.8
Fused/gemination/supernumerary	6	5.9
Leukoplakia	1	1.0
Don't know	3	3.0
Development defect	2	2.0
Fracture teeth	1	1.0
Treatment for case #2:		
Extraction^a^	51	50.5
Pulp therapy/pulpotomy/RCT/pulpectomy	33	32.7
Antibiotics	2	2.0
Referral	2	2.0
Pain meds, antipyric/instructions/diet/	2	2.0
Drainage/incision	2	2.0
x-ray/radiograph/further investigation	6	5.9
Restorative	3	3.0
Diagnosis for case#3		
Avulsion^a^	92	91.1
Trauma/tdi	6	5.9
Extrusion/fracture	2	2.0
Development defect	1	1.0
Treatment for case #3:		
No treatment/assurance	27	26.7
Replantation	13	12.9
No treatment/follow up/oral hygiene/others^a^	32	31.7
Pain meds/antibiotics	4	4.0
Radiograph/x-ray	6	5.9
Space maintainer/nance/denture	12	11.9
Splint	2	2.0
Referral	4	4.0
Diagnosis for case#4		
Molar Incisor hypomineralization/MIH/hypocalcified^a^	78	77.2
Amelogenisis imperfecta	3	3.0
Hypoplastic	3	3.0
Caries/cavities	9	8.9
Pulpitis	6	5.9
Others	1	1.0
Treatment for case #4:		
SSC/crown^a^	65	64.4
Restoration/filling/GI/conservative/fluoride/composite/atraumatic restorative	21	20.8
Pain killer/avoid diet/palliative/Fluoride/oral hygiene instructions	3	3.0
Referral/transfer	3	3.0
Pulp therapy	8	7.9
Diagnosis for case#5		
Early childhood caries/ECC/rampant caries/SECC/caries/decay^a^	93	92.1
Erosion	4	4.0
Amelogenesis/Dentinogensis imperfecta	3	3.0
MIH	1	1.0
Treatment for case #5		
Radiographs/x-rays	3	3.0
Pulpotomy/pulpectomy/pulp therap	16	15.8
Restorations	15	14.9
Extractions^a^	23	22.8
Prevention/fluoride	6	5.9
Diet/stop bad habits	1	1.0
SSC/zirconia crowns	10	9.9
Full mouth rehab/GA	18	17.8
Referral	8	7.9
Diagnosis for case#6		
Caries	4	4.0
Ectopic eruption/Retained incisors/lingual eruption/double row^a^	86	85.1
Normal eruption pattern	3	3.0
Crowding	3	3.0
Malocclusion	1	1.0
Others	3	3.0
Treatment for case #6		
Observation/Assessment/reassurance/leave to exfoliate naturally/wait/no treatment/nothing/follow up/watch	42	41.6
Extract primary teeth/incisors^a^	56	55.4
Others	3	3.0
Diagnosis for case#7		
Functional class III/ant crossbite^a^	65	64.4
Median line shift	1	1.0
Segment/Posterior crossbite	17	16.8
Crowding	4	4.0
Malocclusion/bad occlusion/	13	12.9
Treatment for case #7		
Wait/watch/follow up/observe/no treatment	4	4.0
Removable appliance/z-spring	8	7.9
Remove interference/Cs	3	3.0
Ortho/Ortho referral/functional appliance^a^	60	59.4
Maxillary expansion/reverse headgear/palatal expansion	25	24.8
Diagnosis for case#8		
Molar hypomineralization/Second molar hypomineralization^a^	50	49.5
Caries/cavity	14	13.9
Hypoplasia/hypoplastic	26	25.7
Amelogenesis imperfecta	1	1.0
Pulpitis	4	4.0
Others	4	4.0
Treatment for case #8		
SSC/stainless steel crown/crown^a^	51	50.5
Restoration/filling/GIC/conservative treatment	25	24.8
Pulp therapy	16	15.8
Follow up/nothing	2	2.0
Fissure sealant/fluoride/	2	2.0
Others	3	3.0

a Diagnosis and treatment plan as determined by a gold standard specialist.

**Table 4:** Diagnostic and treatment agreement among respondent dentists.

Case ID	Diagnostic agreement (%)	Treatment agreement (%)
Case #1	54.5%	51.5%
Case #2	65.3%	50.5%
Case #3	91.1%	26.7%
Case #4	77.2%	64.4%
Case #5	92.1%	22.8%
Case #6	85.1%	55.4%
Case #7	64.4%	59.4%
Case #8	49.5%	50.5%

Patterns in treatment decision-making largely mirrored diagnostic findings. Case #4 [molar–incisor hypomineralization (MIH)] showed the highest treatment agreement (64.4%), followed by Case #7 (59.4%). Moderate agreement was recorded for Case #6 (ectopic eruption; 55.4%). The lowest concordance for treatment planning was observed in Case #3 (avulsion; 26.7%) and Case #5 (ECC; 22.8%), indicating marked variability in clinicians' preferred management strategies for these conditions (Figure 3).

## Discussion

This study evaluated the diagnostic reliability and treatment planning agreement among Jordanian dentists who used teledentistry for pediatric dental cases. The results show that teledentistry can assist in initial diagnostic evaluations for certain conditions when high-quality clinical images are available, while also revealing important limitations in treatment decision-making consistency.

High diagnostic agreement was observed for conditions with clear visual presentation, including avulsion (91.1%), early childhood caries (ECC) (92.1%), and ectopic eruption (85.1%). These findings align with previous research showing that photographic-based teledentistry can support accurate initial diagnosis of common pediatric conditions when image quality is optimal (1, 3, 8). Likewise, studies from Saudi Arabia and Kuwait reported acceptable diagnostic accuracy for caries and other oral conditions using teledentistry platforms (9, 10). However, these results should be interpreted carefully. The small number of cases, convenience sampling, and lack of inter-rater reliability testing limit the ability to generalize these findings or to conclude consistently high diagnostic accuracy across broader clinical settings.

The treatment-planning agreement was significantly lower than the diagnostic agreement. While moderate agreement was seen for MIH (64.4%) and functional crossbite (59.4%), agreement was much lower for avulsion (26.7%) and ECC (22.8%). This difference highlights an important limitation of teledentistry in guiding final treatment decisions, especially for conditions that require clinical judgment influenced by patient-specific factors, such as cooperation, pain, disease extent, and restoration feasibility.

The variability observed likely reflects the inherent complexity of treatment decision-making more than differences in provider philosophy alone. Although earlier studies suggest that clinical background and training influence treatment preferences for traumatic injuries and ECC management, this study did not directly measure provider belief systems, clinical experience, or philosophical approaches. Therefore, causal inferences about the source of treatment variability must remain tentative and should be examined in future qualitative or mixed-methods research.

Consistent with international research, teledentistry appears to be more dependable for diagnostic screening than for guiding complex clinical interventions (11). Pediatric Dentists showed significantly higher diagnostic accuracy (82.7% vs. 64.1%) and treatment-planning agreement (54.3% vs. 42.8%) compared to other dental practitioners (*p* < 0.001). This supports regional evidence indicating greater visual diagnostic confidence and digital assessment readiness among Pediatric dental specialists (12, 19). However, when comparing these outcomes with studies from Saudi Arabia, Kuwait, and Iran, it is essential to acknowledge the methodological differences. Many regional studies assessed perception, attitude, or self-reported confidence rather than structured, case-based diagnostic performance. Additionally, differences in national training curricula, case complexity, and imaging protocols may affect observed agreement levels. These differences limit direct statistical comparison but collectively suggest that pediatric specialty training improves readiness for teledentistry-based care.

More than half of the participants (52.5%) reported having no prior teledentistry experience, yet they successfully completed the digital case evaluations. This finding supports regional adoption models, which suggest that baseline experience is not necessary for effective engagement (4, 13, 14). Instead, structured training programs and institutional support seem to be the most important factors driving competence and readiness to adopt.

Saudi data, using the UTAUT model, showed that performance expectancy and social influence significantly predict willingness to adopt teledentistry (13). In contrast, Iranian studies indicated positive attitudes despite limited baseline knowledge (14). These findings support the idea that Jordanian dentists may have considerable latent readiness for teledentistry implementation.

Several obstacles to implementing large-scale teledentistry were identified, including infrastructure issues, regulatory uncertainties, professional liability concerns, and data privacy challenges. These findings reflect previous national assessments in Jordan (5, 15) and align with barriers reported in Saudi Arabia, Kuwait, and other low- and middle-income countries (7, 10, 16, 17).

Without coordinated investments in digital infrastructure, legal frameworks, and standardized clinical protocols, the clinical growth of teledentistry may stay fragmented and underused.

### Strengths and limitations

Strengths of this study include the use of authentic clinical images, a structured case-based evaluation design, and the inclusion of dentists from multiple practice settings. However, several limitations should be acknowledged: *Recruitment through professional social media platforms precluded calculation of an exact response rate and may have introduced self-selection bias. *Potential selection bias favoring digitally inclined clinicians. *Over-representation of pediatric dentists. *Absence of inter-rater reliability testing. *Lack of patient-level outcome validation. These limitations constrain the external validity of the findings and prevent causal interpretation of treatment decision variability.

## Conclusion

Teledentistry holds substantial promise as an effective screening and preliminary diagnostic modality for pediatric dental conditions in Jordan, particularly when high-quality digital images are provided. The consistently high diagnostic accuracy observed—especially among pediatric dental specialists—demonstrates that remote assessment can reliably identify many common and visually distinct pediatric dental problems. However, the variability in treatment decision-making highlights that teledentistry should complement, rather than replace, traditional in-person care when complex management is required.

Pediatric dentists emerged as the most capable group in leveraging teledentistry, positioning them as key leaders in national implementation efforts. Despite limited prior exposure among many participants, their willingness to engage with digital platforms indicates strong readiness for integration, provided that structured training, supportive regulation, and robust technological infrastructure are established.

To strengthen the evidence base and guide policy development, future studies should incorporate inter-rater reliability testing, follow patients longitudinally to evaluate clinical outcomes, and assess cost-effectiveness at the system level. Building this foundation is essential for realizing the full potential of teledentistry as a scalable, equitable, and sustainable component of Jordan's oral healthcare system.

## Data Availability

The original contributions presented in the study are included in the article/Supplementary Material, further inquiries can be directed to the corresponding authors.
