# Supplementary figures and images for: Evaluation of the effectiveness of teledentistry on diagnostic accuracy and treatment planning among Jordanian dentists
Source: Front Dent Med. 2026 Jan 15;6:1705072. doi: 10.3389/fdmed.2025.1705072 (PMC12852331; doi:10.3389/fdmed.2025.1705072)

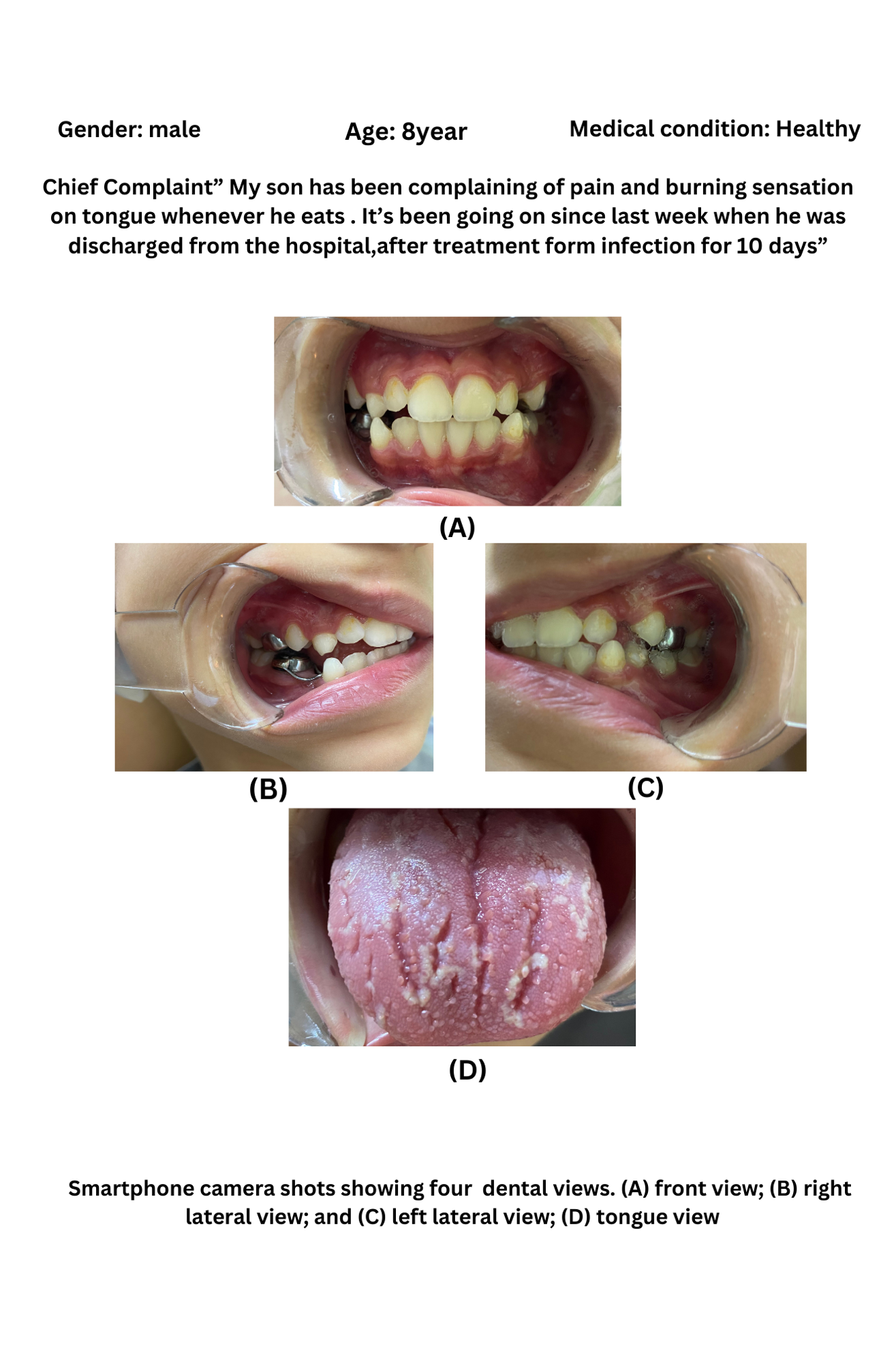


**Case #1**


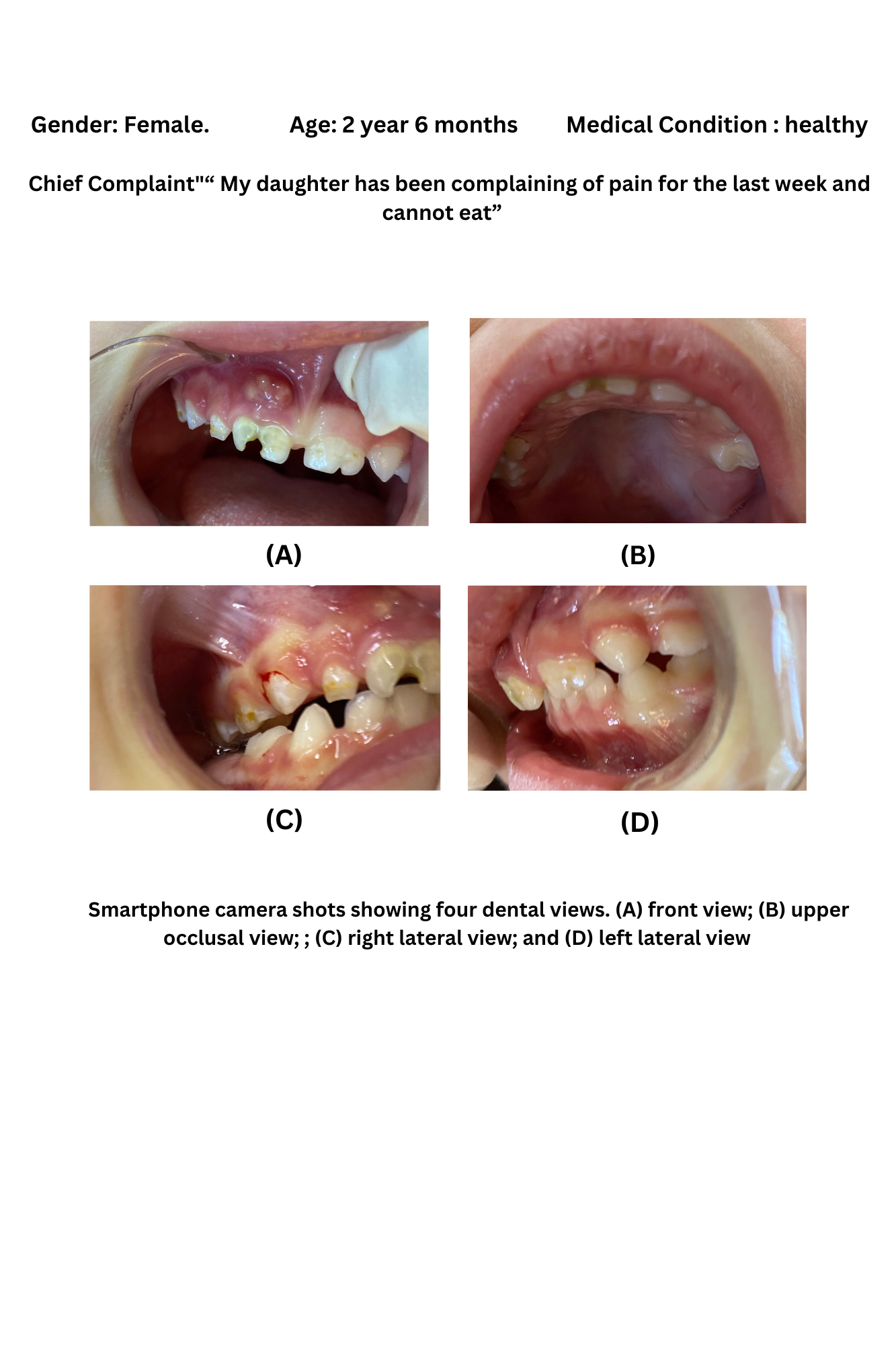


**Case #2**


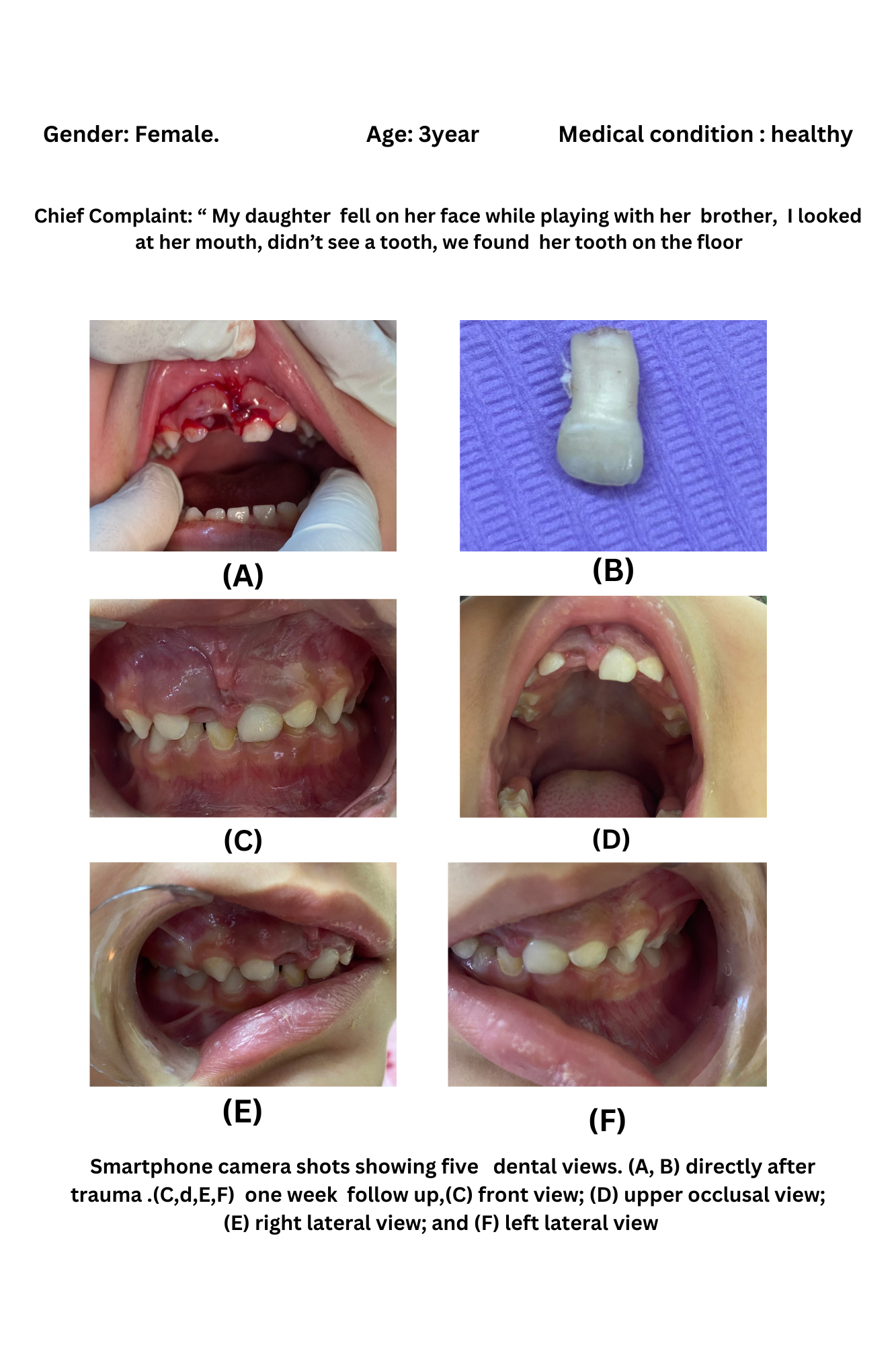


**Case #3**


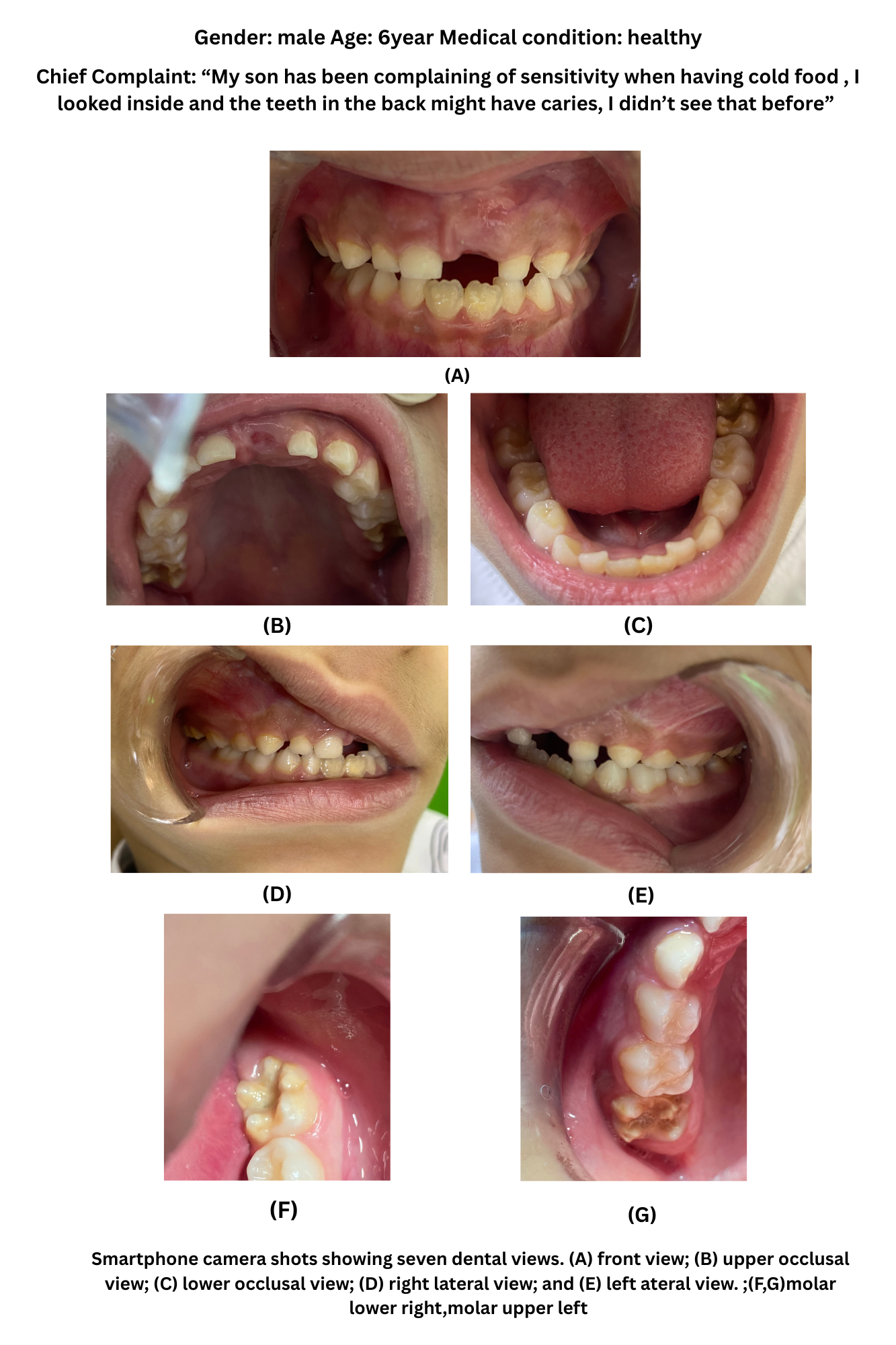


**Case #4**

**Case #5**


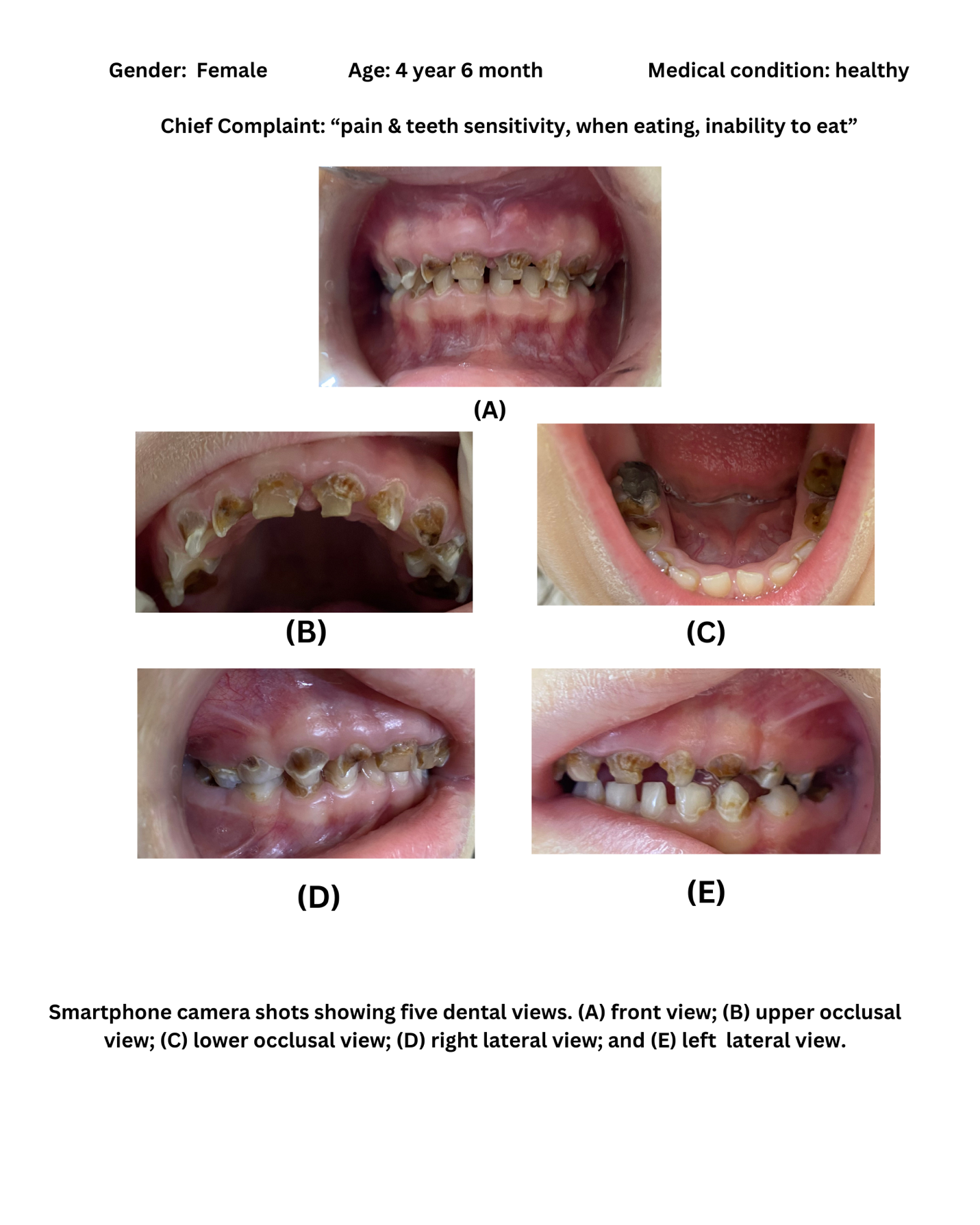


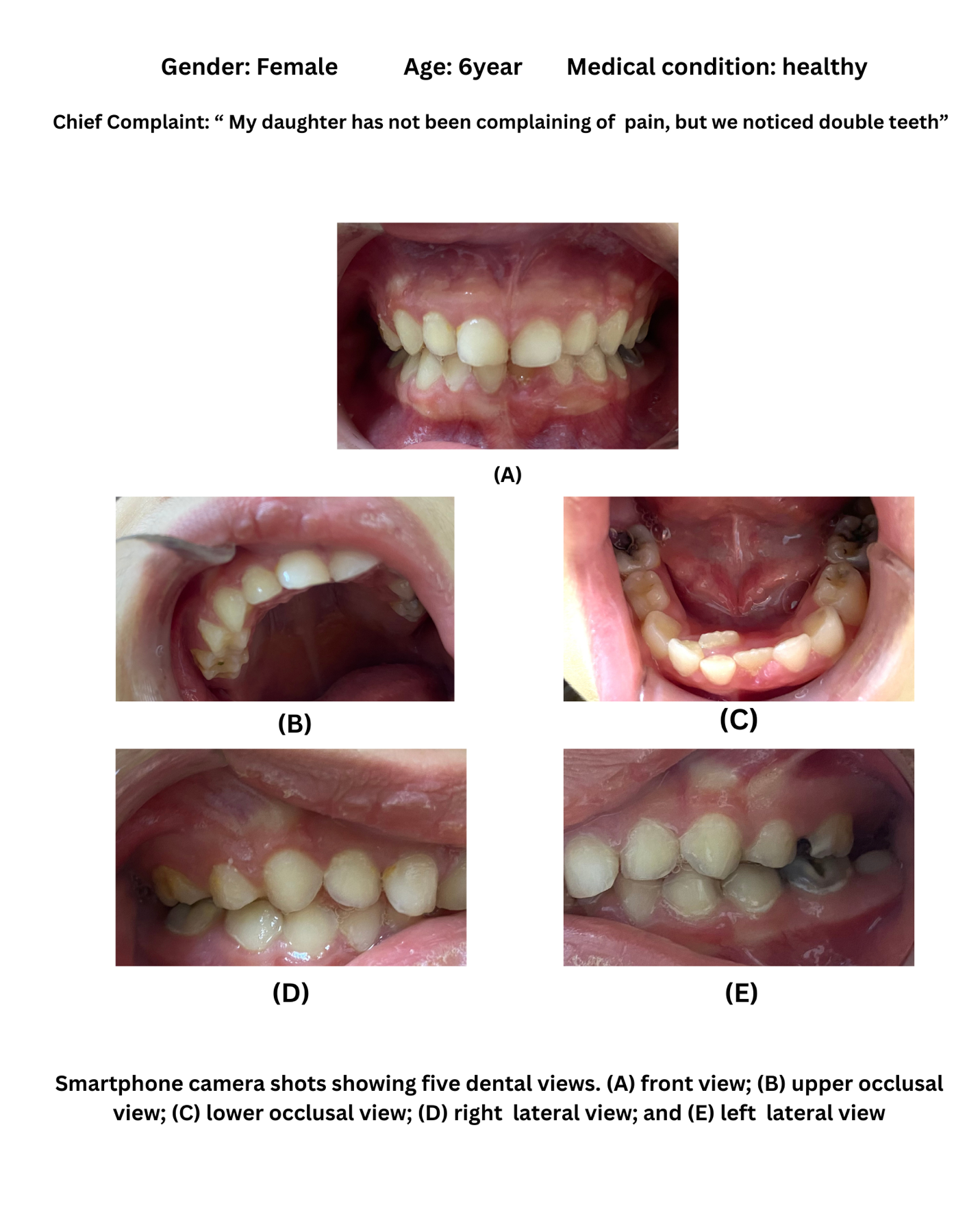


**Case #6**


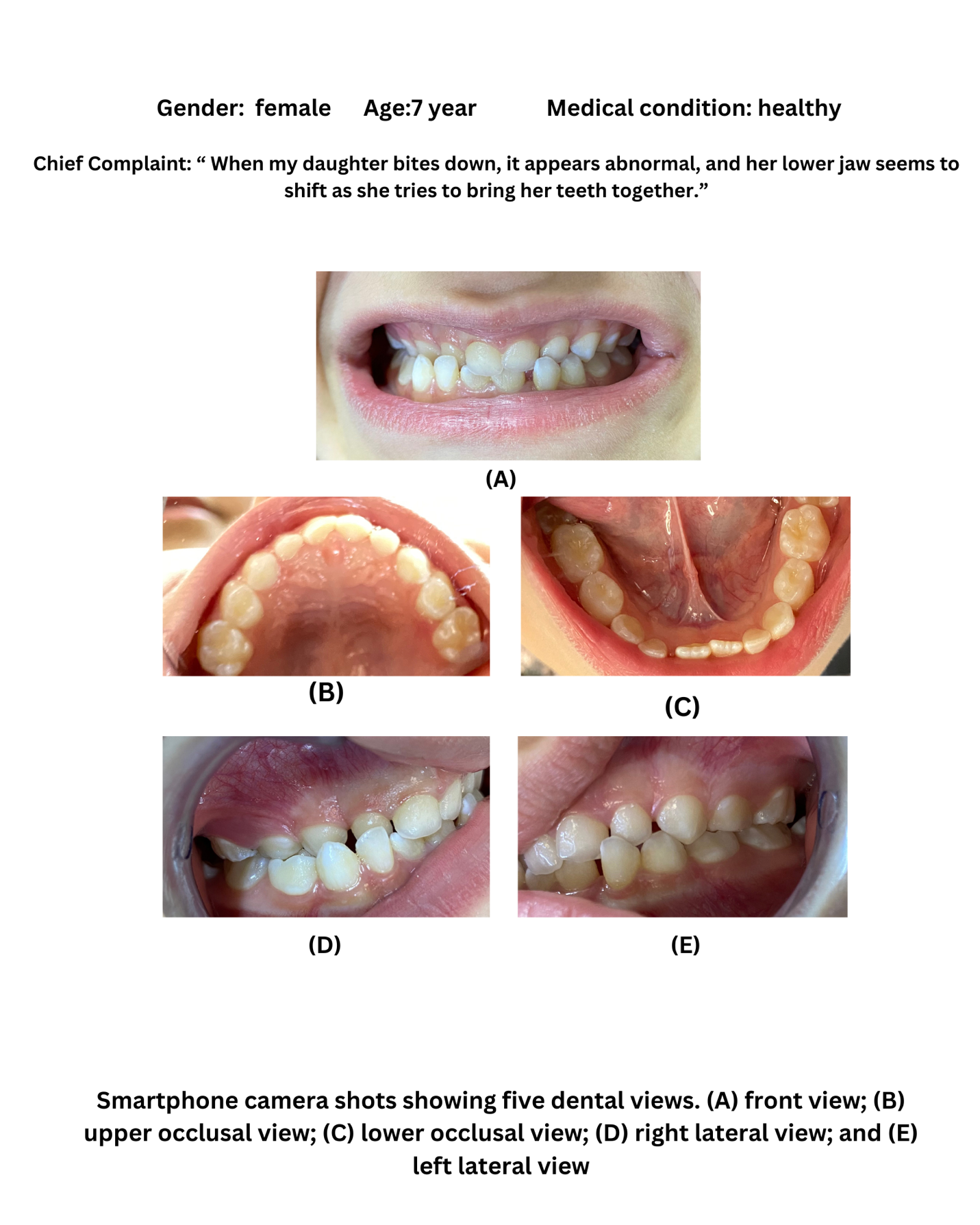


**Case #7**

**Case #8**


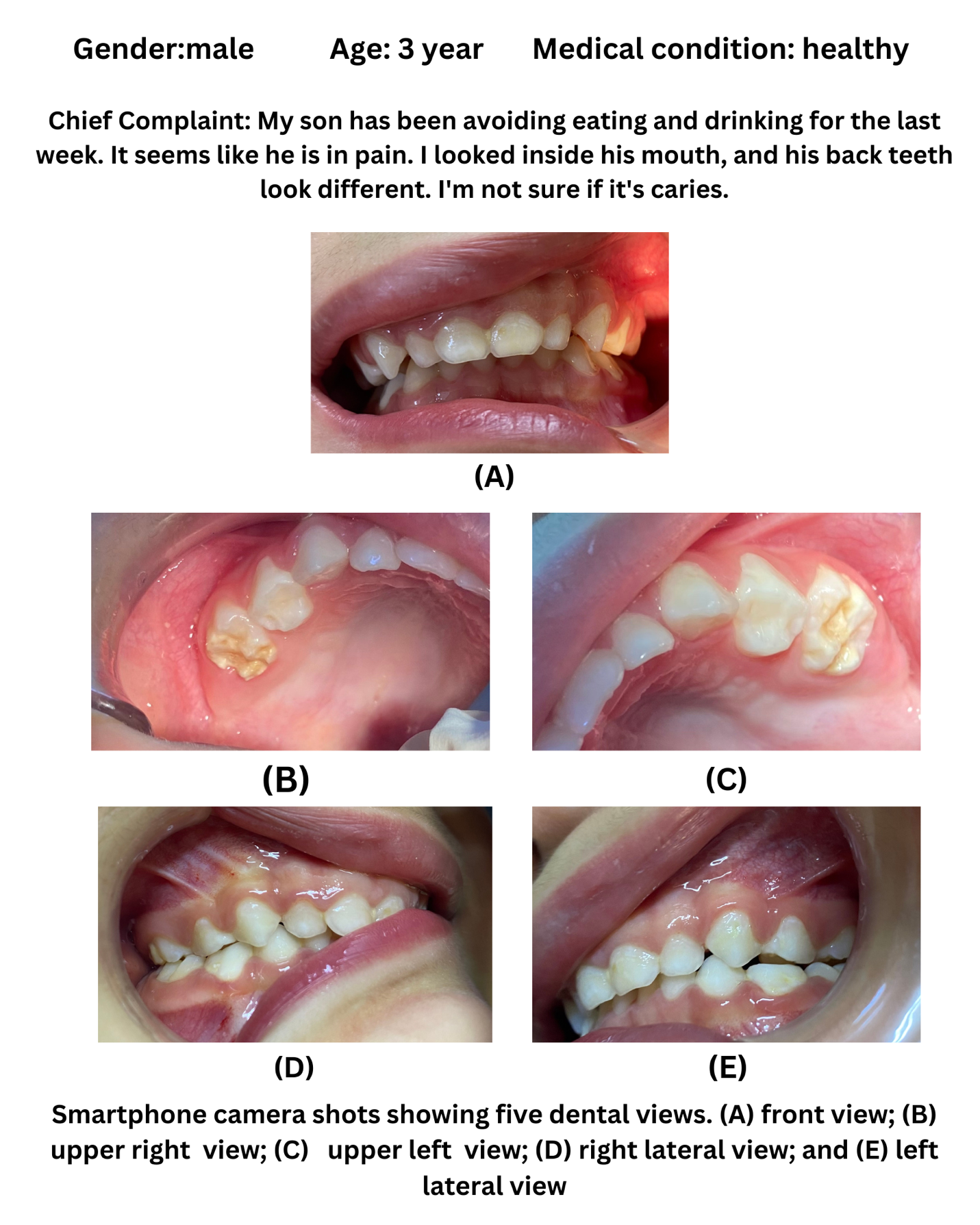

Supplement: Supplementary file 1 [file Table1.docx]
